# Role of Fibroblasts and Myofibroblasts on the Pathogenesis and Treatment of Pelvic Organ Prolapse

**DOI:** 10.3390/biom12010094

**Published:** 2022-01-06

**Authors:** Zeliha Guler, Jan Paul Roovers

**Affiliations:** Department of Obstetrics and Gynaecology, Amsterdam Reproduction and Development, Amsterdam UMC-Location AMC, University of Amsterdam, Meibergdreef 9, 1105 AZ Amsterdam, The Netherlands

**Keywords:** pelvic organ prolapse, connective tissue, extracellular matrix, fibroblasts, myofibroblasts, regeneration, fibrosis

## Abstract

Pelvic organ prolapse (POP) is a multifactorial connective tissue disorder caused by damage to the supportive structures of the pelvic floor, leading to the descent of pelvic organs in the vagina. In women with POP, fibroblast function is disturbed or altered, which causes impaired collagen metabolism that affects the mechanical properties of the tissue. Ideal surgical repair, either native tissue repair or POP surgery using an implant, aims to create a functional pelvic floor that is load-bearing, activating fibroblasts to regulate collagen metabolism without creating fibrotic tissue. Fibroblast function plays a crucial role in the pathophysiology of POP by directly affecting the connective tissue quality. On the other hand, fibroblasts determine the success of the POP treatment, as the fibroblast-to-(myo)fibroblast transition is the key event during wound healing and tissue repair. In this review, we aim to resolve the question of “cause and result” for the fibroblasts in the development and treatment of POP. This review may contribute to preventing the development and progress of anatomical abnormalities involved in POP and to optimizing surgical outcomes.

## 1. Introduction

Pelvic organ prolapse (POP) is a multifactorial condition caused by damage to the supportive structures of the pelvic floor [1], which results in the descent of pelvic organs in the vagina. POP results in problems with micturition, defecation, and sexual functioning, which decreases the quality of life and impairs self-esteem [2,3]. The overall lifetime risk for POP surgery is 10%, and about one out of five surgeries are performed in a patient who has been operated on before [4]. Despite the high incidence of POP, the underlying pathophysiology is still not entirely explained. The major risk factor to develop POP is vaginal delivery [5], along with other factors such as age, overweight, chronic coughing, and genetic predisposition [6,7]. Another important factor is menopause, resulting in a decrease of estrogen and thus vaginal atrophy, causing the thinning and inflammation of the vaginal wall, which disables compensation for birth-related pelvic floor trauma [8]. In women with POP, fibroblast function is negatively affected, which results in impaired collagen metabolism and disturbed extracellular matrix production [5,9,10,11,12,13].

Surgical repair using the patients’ own pelvic support tissues, such as perivesical fascia, known as native tissue repair (NTR) [14], is used as a primary surgical procedure to correct anatomical abnormalities involved in POP. However, NTR is associated with a high risk of anatomical (and functional) recurrence up to 16–29%, as the quality of the tissue that is used is proven to be of inferior quality as the connective tissue is already weakened as a result of POP [15,16,17]. Natural (allografts, autografts, and xenografts) and synthetic implants (permanent and degradable) have been introduced to surgically correct POP [14,18]. Considering anatomical outcomes, many studies have reported the clear superiority of implant surgery over NTR [19,20,21,22]. The use of autografts is limited by morbidity at the harvest site, inconsistent quantity, and quality of the harvested material. Allografts and xenografts have a potential risk of infections. In addition, their performances are worse as compared to synthetic implants. For instance, degradable xenografts such as InteXEN^®^ [23] and Surgisis^®^ (Armitage, Seman et al., 2012) undergo rapid in vivo degradation, and the load-bearing capacity of the remodeled tissue is insufficient [24,25]. Synthetic implants are advantageous to eliminate the risk of transmission of infectious diseases and they can be produced with specific textural, mechanical, and structural features to optimize their performance. Even though implants are initially designed to surgically correct POP and provide mechanical support to the pelvic tissues, ideally they should induce a foreign body response that results in the development of new connective tissue that is mechanically self-sufficient to support descending organs [18,26]. Animal studies in which vaginal implant surgeries were performed indicated that healing response, vascularization, and collagen metabolism are increased, and consequently increase the mechanical properties of the tissue as compared to NTR [27].

Either in NTR or POP surgery with an implant, wound healing and tissue regeneration are highly needed for a successful surgical outcome, which is functional and mechanically self-sufficient pelvic tissue. This can mainly be achieved by a mild and well-orchestrated host response by triggering collagen metabolism without creating fibrotic tissue [28]. The formation of a healthy, functional, new connective tissue will not only improve the surgical outcome, but also contribute to a better tissue repair with less complications. The tissue repair after surgery is related to the characteristics of the connective tissue of POP patients, which is determined by the fibroblasts’ function.

Fibroblasts, having a crucial role in the connective tissue quality such as decreased fibroblast quantity and quality, may contribute to the pathophysiology of POP. On the other hand, fibroblast activity and fibroblast-to-(myo)fibroblast transition play an important role during the healing process and tissue remodeling after POP surgery, and therefore have a significant effect on surgical outcome. (Myo)fibroblast activity can repair the damaged tissue, or in the case of excessive activity, may lead to pathologic fibrotic tissue causing contraction, implant deformation, and may contribute to the pain [29]. The objective of this review is to resolve, if possible, the question of “cause and result” for the fibroblasts in the development and treatment of POP. We will summarize the changes in connective tissue in women with POP, the role of fibroblasts and fibroblast–(myo)fibroblast transition in wound healing and tissue regeneration, and the factors affecting the (myo)fibroblasts’ function. Future perspectives and the gaps in knowledge will be discussed. This review may lead to a better understanding of the role of fibroblasts in pelvic organ prolapse, how to optimize surgical outcomes, and how to prevent the development and progress of anatomical abnormalities involved in POP. 

## 2. Pelvic Organ Prolapse as a Connective Tissue Disorder

### 2.1. Background/Cause

**Figure 1:**
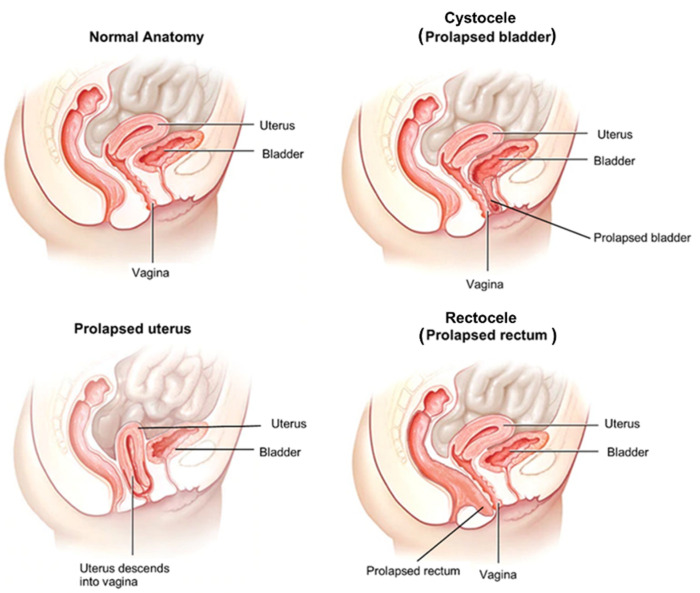
Pelvic organ prolapse. Pelvic organ prolapse (POP) is a multifactorial condition caused by damage to the supportive structures of the pelvic floor. Cystocele (bladder prolapse) is the most common type of prolapse and results in problems during micturition. The bladder’s supportive tissue stretches and sinks against the anterior vaginal wall. Uterine prolapse is a result of the descent of the uterus into the vagina due to the stretches during vaginal delivery and pregnancy. The weight of the uterus may cause prolapse of the other organs. Rectocele (prolapsed rectum) causes a bulge or tear (in severe cases) in the posterior vaginal wall due to the weakening of the tissue.

Pelvic organ prolapse is a multifactorial disease caused by a defect in the connective tissue or pelvic floor musculature, or a combination of the two, which results in the loss of the support provided by pelvic fascia and skeletal muscles [30,31]. Dysfunction of these structures and the decrease in the resilience of the connective tissue alters the support system, and as a result, pelvic organs around the vagina, including bladder, uterus, and rectum descent into the vagina (Figure 1) [32].

The risk factors for POP are vaginal delivery, pregnancy, or other conditions resulting in increased abdominal pressure, such as obesity, chronic coughing, heavy lifting, constipation, and so on. Vaginal delivery is the strongest risk factor for the development of POP as the forces during delivery are significantly higher than the pelvic floor muscles can withstand [33]. Aging is directly correlated with the incidence of POP, as the circulating estrogen levels are drastically decreased in postmenopausal women [34,35]. Estrogen deprivation may cause the development of POP since estrogen has a profound influence on pelvic collagen synthesis and the strength of muscle and connective tissue [34]. Abnormal repair of the injured tissue after the vaginal delivery may lead to changes in ECM in the pelvic floor, consequently affecting the mechanical endurance of the pelvic floor tissue.

Native tissue repair (NTR) by using patients’ fascia is the first option in surgery to correct POP. Due to the higher recurrence rates of NTR, synthetic implants have been introduced to restore pelvic floor function by improving the integrity of existing compromised tissue, which caused POP by inducing a favorable host response. In both surgical treatments, the final goal is to create a mechanically self-sufficient and functional tissue.

The connective tissue of the vaginal wall remodels to adapt to the changes in the biomechanical environment through the extracellular matrix (ECM) metabolism. The remodeling process depends on the quality and quantity of the cellular components of the connective tissue. Before we theoretically explain the changes and remodeling of the connective tissue, we should briefly mention the structure and layers of the vaginal wall in which these changes occur. The vaginal wall is composed of four layers: non-keratinized stratified squamous epithelium, lamina propria of connective tissue, smooth muscle with bundles of circular and longitudinal fibers, and adventitia, which consists of blood vessels, lymphatic ducts, and nerves [36]. The pelvic connective tissue consists of fibrilar components (fibrous elements) such as collagen and elastin, which are the main components of the ECM, of which the metabolism is regulated by the fibroblasts—the main cell type in the vaginal wall (55.19% of the entire cell population in the vagina wall) [9,37,38]. Other important components of the ECM are fibronectin, fibrinogen, vitronectin, laminins, thrombospondin, integrins, and other glycoproteins involved in the cellular adherence process. Integrins are indispensable for ECM metabolism and cellular turnover [39,40]. The fibrilar component contributes the most to the biomechanical behavior and the properties of the pelvic tissues. The quality and quantity of the fibrilar component are regulated through a dynamic, constant remodeling process, which is a precise equilibrium between synthesis, maturation, and degradation of collagen and elastin [41].

Changes in the ECM and collagen metabolism, such as the decreased amount of collagen, increased collagen break-down, or altered ECM remodeling and/or expression of structural proteins resulting from gen-mutations or decreased estrogen levels, may all increase the risk on POP. Another hypothesis is that POP disturbs the repair of injured supportive tissues due to mechanical stress [5,34,42]. The differences in the vaginal connective tissue of the women with POP and no POP resemble the changes occurring in the tissue after injury [41,43]. Increased collagen III and active MMP-9 are typical of tissue remodeling and repair after injury due to overstretching or increased mechanical load. Therefore, observation of increased levels of MMP-9 and collagen III in women with POP in comparison to without POP support this hypothesis. Without a doubt, fibroblasts and (myo)fibroblasts have a key role in the healing and remodeling processes either in the development or in the treatment of POP. In the next section, we take a closer look at the changes occurring at molecular and protein levels in the connective tissue in POP.

### 2.2. Changes in Connective Tissue in POP

Fibroblasts play a key role in collagen metabolism and ECM turnover. There are several changes in the quality and quantity of connective tissue in patients with POP. Either with genetic factors or acquired defects due to mechanical stress, the functionality of fibroblasts differs in prolapsed tissue, which subsequently results in changes in the collagen content in POP. Collagen, as the main component, accounts for up to 80% of the connective tissue. There are 28 types of collagen and the most representative ones on the pelvic floor are collagen type I and III, which are the determinants of tissue strength [44,45]. Collagen I has high stretching ability and resistance to tension, therefore playing a role in strengthening the pelvic structures. Collagen III has flexibility and distension and is predominantly present in the tissues that are subjected to periodic stress [46]. Both collagen I and III are found in the tissue during wound healing [41]. The amount of collagen within the pelvic tissue, as well as the ratio between collagens I and III, are indicative of tissue biomechanics, elasticity, and relaxation ability. A higher or lower I/III ratio is a sign of greater strength or tissue laxity, respectively. The overall collagen content is found to be lower in patients with prolapse in comparison to those without [47]. In addition, it has been shown that the contractibility of the vaginal (myo)fibroblasts is decreased in POP patients, which may result in deficient collagen [5]. In addition, several studies reported an increase in collagen III in the sub-epithelium and muscular layer of the vaginal wall in patients with POP when compared to women without POP, independent of age and parity [10,43,48]. An increased collagen III content could be an expression of tissue repair after overstretching of the supportive connective tissue of the pelvic floor. Since increased collagen III is associated with increased flexibility and distensibility and decreased tensile strength of the pelvic tissues, POP will progress in women with increased collagen type III [43].

The collagen metabolism and ECM turnover are controlled by the synthesis or degradation of ECM components, which is important for maintaining tissue integrity. Degradation depends on the activity of the matrix metalloproteinases (MMP). MMPs are a group of proteolytic enzymes that degrade all ECM proteins. Mainly MMP-2 [12] and/or MMP-9 [43] play a trivial role during the ECM remodeling of the vaginal wall. Several studies indicated that the amount of MMP-2 and MMP-9 is higher in the vaginal wall in women with POP if compared to those without POP [43,49]. Higher MMP-9 expression and increased collagen III in fibroblasts after an injury or after increased applied mechanical load to the pelvic tissues can be an indication of tissue remodeling [50,51] and supports the hypothesis that POP may be related to disturbed and impaired wound healing. To limit connective tissue degradation, the activity of MMPs is regulated by tissue-derived inhibitors of metalloproteinases (TIMPs). TIMP counterbalances the action of MMPs by forming a complex with them, to avoid them binding their substrate, therefore reducing the collagen degradation process [52]. Several POP studies [53,54,55,56] identified an imbalance between MMPs and TIMPs and reported high MMP levels and/or lower TIMP concentrations, which results in an accelerated process of collagen degradation that exceeds its synthesis. Collagen turnover is increased in the fibroblasts from prolapsed tissue, as evidenced by increased MMP-2 and MMP-9 activity and a decrease of the activity of TIMP-1. Therefore, the breakdown of immature newly formed collagen is increased. In general, it was observed that concentrations of poor-quality collagen are higher in women with POP [57].

Another important component of the connective tissue in the pelvic floor is elastin, which allows the tissue to stretch and has passive recoil capabilities (return to its original shape) [58]. The production of elastin is unique among connective tissue proteins. In most organs, elastin biosynthesis is limited to a brief period of development. However, in the female reproductive tract, elastic fiber turnover is continuous throughout the lifespan. The role of elastin is especially important during pregnancy and vaginal delivery as it can accommodate enormous expansion, involution, and stretch [59]. A proper degradation, synthesis, and regeneration of elastic fibers are essential for maintaining pelvic organ support. Decreased elastin mRNA and tropoelastin [60], or increased elastolytic activity [61] in fibroblasts derived from cardinal ligaments of patients with POP, were associated with the progression of prolapse. 

Several factors are affecting the fibroblasts’ activity. The influence of stretch on the fibroblasts, whether as a cause or an effect, should not be overlooked in prolapse. Mechanical stretch disturbs the activity of the fibroblasts by influencing their capacity to synthesize ECM components and maintain their cytoskeleton architecture [37]. Many studies demonstrated the changes in the ECM components of the vaginal fibroblasts based on the applied mechanical forces in an in vitro setting, where the normal and prolapsed vaginal fibroblasts are exposed to mechanical loading [37,41,49]. These observations suggest that for women with mild symptoms and severe anatomical abnormalities, the benefit of surgery is in order to avoid continued stress on the prolapsed tissues, which deteriorates the collagen quality and the recovery potential.

As we discussed, POP might result from a disturbed healing process after trauma. The disease itself affects the stiffness and composition of the ECM. The changes in connective tissue during the development of POP resemble scar tissue by being stiffer and having increased amounts of collagen type III relative to collagen type I protein. Therefore, vaginal fibroblasts might recognize the resulting POP-ECMs after an injury as scar tissues that need to be remodeled. In the case of treatment, during the vaginal wall reconstruction by NTR with autologous fascia graft or implantation of a scaffold, the vaginal microenvironment and the ECM stiffness change. Either the NTR procedure or the presence of a stiffer implant than the POP tissue triggers the wound healing process and promotes fibroblast–myofibroblast differentiation. Understanding the mechanisms of tissue repair and the involvement of fibroblast–(myo)fibroblast transition in the healing process can provide a rational basis for disease modeling and innovative solutions in vaginal reconstruction for POP.

## 3. Role of Fibroblasts in Wound Healing and Tissue Regeneration

**Figure 2:**
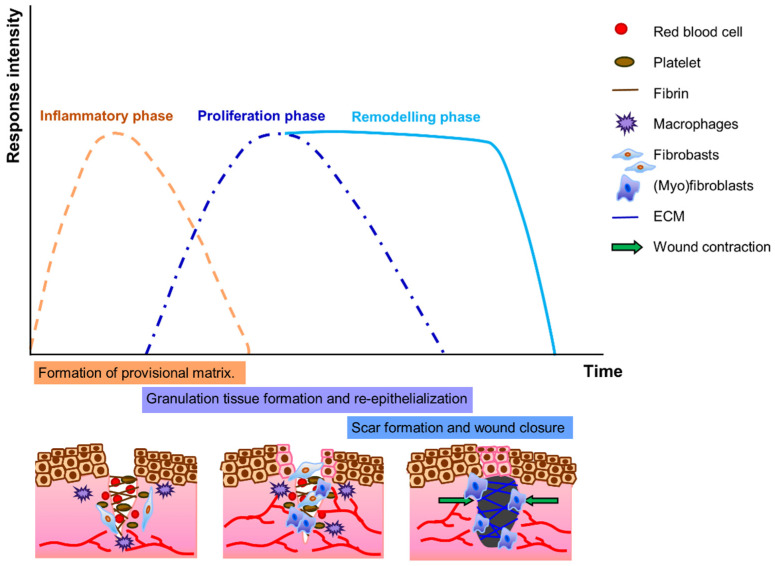
Wound healing phases. In the inflammatory phase, fibroblasts synthesize a provisional matrix consisting of fibrin. Platelets release multiple chemokines that recruit inflammatory cells, including macrophages, as well as fibroblasts. In the proliferation phase, a new capillary network develops and contributes to the proliferation of fibroblasts. Fibroblasts are transient to (myo)fibroblasts, which encourages the synthesis of ECM for maturation of granulation tissue. During the remodeling phase, scar tissue is formed by ECM remodeling, replacement of collagen III by collagen I, and an increase in elastin in the tissue. (Myo)fibroblasts contract the scar tissue and the wound is closed.

Damaged pelvic floor tissues can only recover themselves by wound healing and regeneration by fulfilling two urgent tasks: (1) establishing tissue hemostasis, fighting inflammation, and discarding debris, which is carried out by immune and inflammatory cells, and (2) providing mechanical tissue coherence by forming a scar, which is the task of fibroblasts and so-called myofibroblasts [11]. In normal healing, three overlapping stages follow tissue hemostasis: (i) inflammatory, (ii) proliferation (the development of granulation tissue), and (iii) remodeling (including maturation, scar formation, and re-epithelialization) phases (Figure 2) [62]. Fibroblasts are critical in all three phases, and they are indispensable in determining how well the wound will ultimately heal as they regulate immune response, deposition, and remodeling of ECM components by secreting many cytokines and matrix proteins and by wound contraction [63]. In the inflammatory phase, fibroblasts synthesize a provisional matrix, consisting of fibrin, fibronectin, and glycosaminoglycan hyaluronic acid, that eventually forms a clot [64]. Platelets present in the blood clot release multiple chemokines that recruit inflammatory cells, neutrophils, and macrophages, as well as fibroblasts. In the proliferation phase, which is the most critical stage for proper healing, a new capillary network develops and delivers nutrients to the wound and contributes to the proliferation of fibroblasts. During this phase, fibroblast-to-(myo)fibroblast transition occurs and determines the fate of the tissue repair. We will explain this transition in more detail. In the granulation tissue, fibroblasts are activated and acquire α-SM actin expression and become myofibroblasts. (Myo)fibroblasts feature high cellular contractility and encourage the synthesis of matrix proteins for maturation of granulation tissue to promote faster closure of the wounds in the next phase of healing [65]. During the remodeling phase, proteolytic enzymes, essentially MMPs and TIMPs, play a major role in modifying the collagen III-rich ECM that is synthesized during the proliferation phase [66]. ECM synthesis in the remodeling phase is reduced, collagen III is replaced by collagen I, and elastin increases in the tissue. (Myo)fibroblasts are responsible for wound contraction via muscle-type action, which is facilitated by their cytoplasmic microfilaments (actin-rich stress fibers) and the expression of alpha-smooth muscle actin (α-SMA). Together with cell-to-cell and cell-to-matrix junctions, collagen I and III fibers in the ECM are pulled toward the cell body, which leads to a reduction of granulation tissue. As a result of pulling of collagen fibers and remodeling of ECM, the wound is contracted [67]. Remodeling is sufficient and completed when the tissue regains its mechanical coherence, but not necessarily its functionality. The α-SMA expression becomes downregulated when the contraction is over, and the number of (myo)fibroblasts, together with vascular cells, are dramatically reduced by apoptosis when the tissue is sufficiently remodeled and repaired, and eventually the wound is closed [68]. 

Throughout the wound healing process, (myo)fibroblasts are embedded in the ECM, in which they secrete at the injury site, and a complex and interactive dialogue exists between (myo)fibroblasts and their microenvironment which leads to cell differentiation, proliferation, ECM remodeling, and, in the case of “normal” wound healing, also to apoptosis [65,68]. Fibroblast–(myo)fibroblast transition and (myo)fibroblast phenotype regulated by various factors are critical for tissue regeneration. The altered or disturbed microenvironment around (myo)fibroblasts can lead to repairing defects and fibrosis. By keeping in mind the role of fibroblast activity in connective tissue quality, the transition of fibroblasts to (myo)fibroblasts and their elimination is critical for proper tissue healing and regeneration without a fibrotic response.

### 3.1. Fibroblast–(Myo)Fibroblast Transition

**Figure 3:**
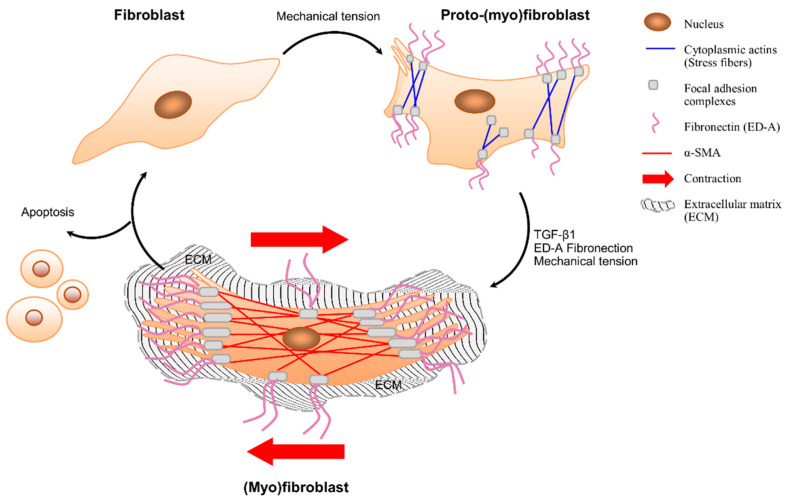
Fibroblast–(myo)fibroblast transition. Fibroblasts do not contain stress fibers or form adhesion complexes with their extracellular matrix. Fibroblasts become activated by mechanical stress and/or cytokine release and differentiate into “proto-(myo)fibroblasts” that form cytoplasmic actin-containing fibers, cell-matrix connections, and fibronectin—including ED-A splice variant (at the cell surface). Proto-(myo)fibroblasts can form contractile forces. In the presence of mechanical stress, TGF-β1 and ED-A promote the transition of proto-(myo)fibroblasts to (myo)fibroblasts, which express α-SMA, larger cell-matrix connections, and extensively develop stress fibers. (Myo)fibroblasts produce ECM and have the ability to contract the surrounding tissue. (Myo)fibroblasts are cleared by apoptosis or may return to a quiescent fibroblastic phenotype after deactivation.

Differentiation and transition of fibroblasts to (myo)fibroblasts occurs in a two-step process, including (1) the formation of proto-myofibroblasts and (2) the development of myofibroblasts (Figure 3). First, fibroblasts develop contractile bundles called stress fibers to re-populate damaged tissues [13]. This phenotypic change in fibroblasts occurs in response to changes in composition, mechanical tension, and locally released cytokines [13]. This activated phase of fibroblasts can be called “proto-myofibroblasts” as they can be discriminated from quiescent fibroblasts as they have contractile apparatus consisting of cytoplasmic actins that generate small traction forces [13]. Most of the knowledge on the formation of proto-myofibroblasts comes from in vitro studies. The role of mechanical tension on the formation of proto-myofibroblasts and myofibroblasts was demonstrated by culturing fibroblasts on collagen substrates with different mechanical properties that can mimic the interaction between fibroblasts and ECM. These collagen substrates can reflect the tethered structure of the tissues in which cell contraction increases the stiffness of the surrounding matrix. As a result of increased mechanical tension, fibroblasts acquire the proto-myofibroblast phenotype and form stress fibers, adhesion complexes, and fibronectin fibrils. The proto-myofibroblast phenotype is maintained when there is a continuous interaction between cell-generated stress and the reaction of a substrate that is sufficiently stiff to resist this force [69]. This is also true in the case of vaginal fibroblasts isolated from POP patients as well as healthy fibroblasts. POP fibroblasts have higher mechano-sensitivity and reveal active responses from cytoskeletons exhibiting higher F-actin, α-tubulin, or vimentin expression in response to the tension existing on the culture substrate [70]. 

The second step in the fibroblast–myofibroblast transition takes place due to the increased mechanical stress in the ECM as a result of the remodeling activity of proto-myofibroblasts, which leads to differentiation of these cells to “myofibroblasts” [13,71]. The contractile nature of myofibroblasts has some similarities to smooth muscle cells, despite the differences in expression of cytoskeletal features. Myofibroblasts can, depending on the experimental or clinical situation, express other smooth muscle-related contractile proteins, such as smooth muscle myosin heavy chains or desmin; however, α-SMA is the most reliable marker of the myofibroblastic phenotype. 

In addition, myofibroblasts have cell-to-matrix (fibronexus) junctions which allow them to make strong attachments, contract and remodel the ECM, and thus transduce the mechanical forces in the tissue [71]. As a hallmark of connective tissue remodeling, stress fibers and α-SMA are incorporated and enhance the contraction [72]. The expression of α-SMA is controlled by growth factors such as transforming growth factor (TGFb1), specialized ECM proteins such as the splice variant form of fibronectin (ED-A fibronectin), and the mechanical microenvironment. TGFb1 increases most collagens in the ECM and inhibits their degradation, thereby promoting tissue regeneration. Their concurrent action of mechanical stress that develops within the tissue and growth factors such as TGF-b1 results in a positive feedback loop, in which tension facilitates TGF-b1 production and/or activation of a-SMA expression, which, in turn, increases force generation and tension development [13]. Along with the other factors, these changes provide a continued formation and sustained function of myofibroblasts. 

Prolapsed connective tissue resembles the characteristics of scar or granulation tissue by being stiffer [73,74] and having a lower collagen I/III ratio [10,43,48,75] as compared to healthy tissue. The altered, abnormal ECM in the prolapsed tissue may contribute to the maintenance of POP by regulating vaginal fibroblast to myofibroblast differentiation [49,70,76]. Ruiz Zapata et al. investigated how matrix stiffness and composition regulate myofibroblast differentiation in vaginal fibroblasts by employing a series of matrices with known stiffnesses, as well as vaginal ECMs, in combination with vaginal fibroblasts from POP and healthy tissues. They reported that α-SMA production was positively correlated with stiffness. Differentiation of fibroblasts to myofibroblasts as evidenced by the increase in α-SMA and collagen gene expression was more pronounced in cells seeded on POP-ECMs which were stiffer than ECMs derived from healthy tissues. It was concluded that when vaginal fibroblasts are seeded on prolapsed ECM, they differentiate into myofibroblast as they recognize surfaces as granulation tissues that need to be remodeled [77]. Prolonged exposure of vaginal fibroblasts to an abnormal prolapsed matrix results in phenotypical changes. Weng et al. applied mechanical stretching to vaginal fibroblasts and found that intercellular spaces between stretched fibroblasts became wider as the fibroblasts became smaller, irregular, and longer due to the changes in the interconnected cytoskeletal system. Healthy vaginal fibroblasts expressed more cytoskeletal components when a cyclic mechanical load (such as stretching) was applied, however a decrease in expression of the same components was seen in the case of POP fibroblasts. This may suggest that POP fibroblasts have a lower tolerance for stretching forces, as their mechanical stretching properties have already reached their limits due to the long-term and excessive stretching load caused by pregnancy, delivery, and intra-abdominal pressure [70]. Therefore, in the case of POP fibroblasts, stretching resulting in overloading may destroy fibroblasts’ cytoskeletal system and metabolic function. 

The cytoskeletal system and intercellular or cell-to-matrix junctions are not only responsible for cell shape but also for the transduction of mechanical signals between cells and contraction of the tissue. Meyer et al. investigated the contraction role of the vaginal myofibroblast in patients with POP. They compared the contractile forces of the myofibroblasts from young primiparae women who had given birth several years ago prior to POP surgery with older women undergoing POP surgery. According to a myofibroblast-mediated collagen gel contraction assay, vaginal myofibroblasts of young women showed better contraction forces (more rapidly and stronger) than young women with severe uterovaginal prolapse. When they compared the contractile forces of myofibroblasts in young primiparous women with the presence or absence of some degree of uterovaginal prolapse, they found that the genital myofibroblasts of women with uterovaginal prolapse reveal a poor contraction [76]. Therefore, POP fibroblasts or myofibroblasts after differentiation may not be able to restore the ECM accurately or provide sufficient contraction, thus creating a positive feedback loop, where the matrix and cells influence each other in a vicious cycle. This vicious cycle can lead to lower strength, increased stiffness, and eventually, more tissue damage, contributing to the worsening of POP [70,76]. However, the repair and regeneration of the pelvic floor can be promoted by changing the microenvironment with the implantation of a scaffold that promotes a host response.

Many different biomaterials have been shown to activate macrophages, which in turn contribute to the generation of myofibroblasts by producing TGF-β1 [11]. Even though this may induce fibrosis in exaggerated cases, such as highly stiff old-generation PP meshes, there are attempts to promote tissue regeneration by applying tissue-engineered strategies. The type of material, mechanical and textural properties such as pore shape, and the size of the implants play an important role in cellular and host responses [9,78]. Implants with larger pore sizes and porosity induce a more favorable host response with increased tissue ingrowth, greater vascularization, and less fibrous tissue as compared to implants with smaller pores. Larger pores allow cellular infiltration and passage of the immune cells, which may reduce the risk of chronic infection [79,80]. In addition, higher porosity results in lightweight implants that are less prone to infections and erosions [81]. Our recent in vitro study illustrated that vaginal fibroblasts isolated from POP patients seeded on poly-4-hydroxybutyrate (P4HB) scaffolds in different construct designs generate a more favorable cellular proliferation and collagen deposition than fibroblasts seeded on polypropylene (PP) meshes [9]. In addition, we demonstrated that the knitting pattern of the scaffolds determines the mechanical and degradation properties of the implant and fibroblast functions. The study of Vashaghian et al. [82] investigated the effect of scaffold properties of polycaprolactone (PCL) and polylactic-co-glycolic acid (PLGA) nanofiber matrices on ECM produced by human fibroblasts. They found that the quality and the type of ECM produced by the fibroblasts were changed with the pore size of the matrices, as indicated by the higher collagen amount in matrices of 1 μm fiber size compared to 8 μm. 

Degradable materials can provide an advantage in terms of reducing clinical complications in the long term [28]. A gradual implant degradation induces functional vaginal tissue with mechanical integrity and results in a moderate or decreased inflammatory response over time [28,83]. Poly(lactic acid) (PLA) dramatically promoted the formation of ECM by increasing the attachment and metabolic activity of fibroblasts on PLA meshes as compared to other meshes: AlloDerm (LifeCell Corp., Branchburg, NJ, USA), cadaveric dermis, porcine dermis, polypropylene, sheep forestomach, and porcine small intestinal submucosa (SIS) [84]. Silk fibroin (SF) appeared to be successful in promoting the growth of fibroblasts into the scaffold and inducing healthy tissue formation in defective areas such as the abdomen, vagina, and pelvis [85]. Polylactic-co-glycolic acid/polycaprolactone (PLGA/PCL) electro-spun scaffolds promoted the differentiation and proliferation of myofibroblasts and enhanced the integration of host cells and scaffolds [82]. PCL/gelatin induced accumulation of cells to the scaffold and the deposition of the matrix [86]. Poly(l-lactide-co-caprolactone) (PLCL) and fibrinogen electro-spun scaffolds promote initial angiogenesis and tissue remodeling at the graft site, and significantly improve the symptoms of patients with prolapse [87]. Our recent in vivo study [28], where we investigated host response to P4HB implants, showed a lower inflammatory response and higher tissue remodeling relative to PP implants 6 months after vaginal implantation into sheep. In addition, we observed milder myofibroblast differentiation in P4HB at 6 months as compared to PP. The mild host response to the P4HB implant was attributed to the exceptionally low membrane stiffness. The stiffness of the implant strongly correlates with implant weight and porosity [14]. Constant applied load after implantation can cause an increase in the implant stiffness, collapse of the pores, and therefore a reduction of porosity, which in turn may lead to vaginal degeneration and eventually exposure [28,88,89]. Therefore, studies on tissue-engineered implants where a cyclic loading is applied to mimic the pelvic floor are performed. Gentle cyclic loading upregulated genes involved in the synthesis of collagen I, III, and elastin matrix remodeling (α-SMA, TGF-β1, and MMP-2) [90].

Another way to trigger fibroblast proliferation and differentiation to myofibroblasts is by adding growth factors to the vaginal microenvironment. Epidermal growth factor (EGF) and basic fibroblasts growth factor (FGF) [91], or connective tissue growth factor (CTGF), as a downstream signal of TGF-b1, is a newly discovered growth factor that can stimulate the proliferation of fibroblasts and collagen deposition and can promote proliferation, migration, and differentiation of fibroblasts to myofibroblasts [92]. The endothelin-1 (ET-1) system regulates (myo)fibroblast contraction in wound healing. Therefore, regulation of the ET-1 system through specific receptors may attenuate myofibroblast contraction and tissue strength [5]. Estrogen plays an important role in the development of POP. It is observed that low estrogen levels in women who undergo vaginal surgery affect the surgical outcome [34], and women who have continuously low estrogen levels exhibit delayed recovery due to impaired wound healing [93]. Therefore, adding estrogen to the surgical site promotes pelvic floor tissue repair by facilitating fibroblast proliferation and collagen synthesis [92,94]. These bioactive factors can also be useful at the implantation site of POP surgery as well as incorporating them in pelvic floor implants. 

### 3.2. Unpaired Healing—Fibrosis

**Figure 4:**
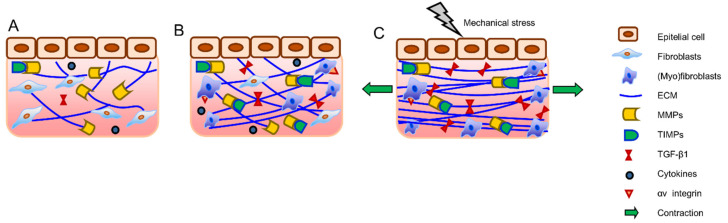
Fibrosis. ECM metabolism and turnover is dependent on the function of fibroblasts, MMPs, TIMPs, and the local tissue environment (**A**). TIMPs regulate MMP activity and directly inhibit ECM degradation, and the increase of TIMP function and release of TGF-β1 results in excessive ECM deposition (**B**). In addition, activated (myo)fibroblasts express integrin receptors that connect TGF-β1 and ECM components, resulting in fibrotic tissue with denser and straighter ECM fibers, which eventually leads to overall higher tissue stiffness. At this point, even smaller mechanical stresses applied to the fibrotic tissue will cause the release of more TGF-β1 (**C**).

(Myo)fibroblast activity and ECM remodeling are designed to regenerate functional tissue, which is, as explained before, a highly regulated process. When the job of (myo)fibroblasts is complete, they should be eliminated from granulation tissue. The granulation tissue with its dense content of (myo)fibroblasts creates the ECM, and apoptosis should result in decreased proteases, therefore forming a hypocellular scar [95]. However, in pathological conditions, the normal wound healing process is dysregulated, which results in decreased or discontinued apoptosis. Therefore, if myofibroblasts persist and continue to remodel the ECM, it eventually causes tissue contraction [11,96]. Vasin et al. indicated that fibrosis of the connective tissue in the vaginal wall predominates in POP. They found an increase in the content of type III collagen, a decrease in the amount of type I collagen, and elastic fibers with significant fragmentation in the scar areas [97]. The exact reason and mechanisms of induction or inhibition of apoptosis are not well-understood, however, apoptosis in myofibroblasts is thought to be regulated by a reduction in the local growth factors that drive and sustain myofibroblast differentiation. In particular, local concentrations of TGFβ-1 and endothelin-1 play a role in (myo)fibroblast survival protein kinase B (AKT) activation [98]. TIMPs also control the altered transforming growth factor (TGF) β signaling, inflammation, or the number of myofibroblast-like cells, which all potentially alter ECM turnover, leading to increased ECM deposition. Increased TIMP levels result in ECM accumulation or fibrosis (Figure 4) [99]. The focal adhesion complex component (Hic-5) [100] and excessive LOX expression [101] are shown to contribute to fibrosis by maintaining the myofibroblastic phenotype [100]. LOX expression in fibroblasts corresponds with their differentiation to active myofibroblasts, which plays a role in fibrotic disorders. Although the key function of LOX is to contribute to crosslinking of collagen and elastin fibers, LOX proteins also have a pro-apoptotic effect that leads to cell death [81]. Vaginal fibroblasts from POP patients have been shown to express lower transcript levels of LOX both in vitro and in vivo [102,103]. This suggests that lower LOX expression in POP vaginal fibroblasts may be contributing to the pathophysiology of POP by contributing to altered connective tissue and also to fibrosis by reducing the apoptosis of myofibroblasts. 

The changes in mechanical forces and signaling through integrins and TGFβ-1 are also playing a critical role in fibrosis [71,100]. Stimulation of myofibroblasts by TGF-β1 itself is affected by mechanical forces within the damaged or fibrotic tissue. TGF-β1 is released in its latent form from a variety of inflammatory cells and platelets in the microenvironment of damaged or fibrotic tissue. Fibroblasts in normal tissue do not express or present integrin receptors that bind and activate latent TGF-β1. However, during tissue repair and fibrosis, activated myofibroblasts express αv integrins that connect the contractile actin/myosin cytoskeleton to latent TGF-β1 [104]. Due to the mechanical stress and myofibroblast contraction, latent TGF-β1 becomes activated. As a result, excessive remodeling and deposition of collagen by myofibroblasts results in overall higher tissue stiffness. Since ECM fibers are straighter, even smaller strains applied to the fibrotic ECM externally, or by residing myofibroblasts, will be sufficient for the release of active TGF-β1. The feedback mechanism assures a persistent fibrotic activity by the myofibroblast, and straining and/or stiffening of the ECM can increase the availability of TGF-β1 (Figure 4C) [71,105]. It was found that POP vaginal fibroblasts express higher mRNA levels of α- and β-integrins [106] which may implicate altered cell–matrix interactions, the potential difference in contractile properties of the myofibroblasts, and the mechano-transduction mechanism in POP. 

Introducing a biomaterial to damaged tissue, such as POP, in this case, can trigger the normal healing process and result in mechanically stronger tissue due to optimal connective tissue remodeling. However, the mechanical load of the intra-abdominal forces on the vagina and surrounding tissues, together with the unpaired implant properties and altered fibroblast function, can induce fibrosis. We already mentioned that the fibroblasts in prolapsed tissues are exposed to altered ECM and therefore, they may display abnormal phenotypes, including decreased proliferation, and altered patterns of cytokine release, as well as abnormal MMP and TIMP activity [106] or apoptosis after differentiation to myofibroblasts [107]. Reconstructive surgery by native tissue or implant surgery can restore pelvic floor function. Implantation of the scaffold results in the release of platelet-derived growth factor (PDGF), vascular endothelial growth factor (VEGF), and TGFβ, which subsequently results in roaming of fibroblasts to the implantation site. Fibroblasts can appear from early to late post-implantation and deposit collagen I and III to repair the damaged tissue [108]. However, the implant properties, such as porosity, construction type, degradability, and mainly stiffness [109,110], can alter the host response and result in excessive and redundant production of collagen and a higher collagen I/III ratio, which eventually leads to a fibrotic response [78,111]. To trigger proper healing without creating a fibrotic response is the key in reconstructive POP surgery. 

Old-generation PP meshes had a very high density (weight per surface), which implies that they were stiff and had incompatible mechanical properties with the vaginal wall. Therefore, they resulted in a disturbed host response, fibrosis, and finally exposure [4,112]. PP meshes have been modified to lightweight and microporous structures with high porosity to reduce the fibrotic response [78,113], which has resulted in fewer adverse events [114,115]. Even though the threshold values for tissue composition in terms of myofibroblasts, collagen and elastin amount, and MMP and TIMP levels are not determined, a moderate host response is desired after vaginal implantation for the success of the surgery and to eliminate the adverse event. Our research group has been working on the development of a biodegradable material, poly-4-hydroxybutyrate (P4HB), as a candidate material for pelvic floor surgery [9], with the hypothesis that a delayed absorbable implant will provide mechanical support while being gradually replaced by functional connective tissue. We evaluated the host response and biomechanics of the fully degradable P4HB implant as compared to PP mesh in sheep [28], a validated animal model for POP surgery [116,117]. P4HB implants generated a moderate host response, which is demonstrated by increased tissue remodeling, low myofibroblast differentiation, and formation of well-organized collagen over time, compared to the PP mesh. Hympánová et al. compared the host response of the PP mesh, electro-spun biodegradable ureidopyrimidinone-polycarbonate (UPy-PC), and electro-spun non-degradable polyurethane (PU) mesh in comparison with native tissue repair (NTR) in a sheep vaginal model. Similar connective tissue composition, vascularization, and innervation were observed after implantation of all materials. The (myo)fibroblast differentiation and inflammatory response to electro-spun implants were mild and comparable with PP. However, PP exhibited higher myofibroblasts as compared to NTR at 6 months post-implantation [118]. Other degradable materials such as polylactic acid (PLA) [119] and modified PCL [101] have also been studied in comparison to PP implants for their potential in vaginal surgery. Tayrac and colleagues did not report a significant difference in the biocompatibility of PLA compared to PP. Modified PCL required a longer time to provide tissue reinforcement and resulted in a vigorous inflammatory response, which may induce fibrosis in the long term. 

ECM-mimicking scaffolds can also create a more optimized host response as they mimic the tissue architecture which regulates the vaginal microenvironment. Electro-spun implants for example can create an ECM-like topography that can provide larger surface areas, enabling protein adsorption and cellular binding sites. PLCL, with its biocompatibility and elastic modulus matching to vaginal native tissue, has been used in pelvic floor tissue engineering. PLCL blended with fibrinogen resulted in a less fibrotic response by calming down the aggressive foreign body reaction as compared to PP mesh [87]. However, the porosity and the pore size should be carefully set as the smaller pores can trigger exaggerated host response and fibrosis [115,120].

Releasing mechanical stress or reducing stiffness can prevent fibrosis and reduce α-SMA expression and myofibroblast contraction [121,122]. In addition, targeting cytokines, growth factors, and pathways that induce myofibroblastic transition, their maintenance, or contractile function can be a way of balancing between normal healing and fibrotic response. 

## 4. Conclusions

In this review, we explained the role of fibroblasts and fibroblast–(myo)fibroblast transition in POP, which is a connective tissue disorder. Changes in the pelvic connective tissue can “cause” development of POP and/or at the same time, these changes can occur as a “result” of POP. Risk factors for POP, such as vaginal delivery, aging, weight, coughing, and genetic predisposition, lead to changes in the collagen metabolism by affecting the fibroblast function [6,9,34]. In addition, the same risk factors, such as vaginal delivery, can create an increase in intra-abdominal pressure and a great stretch on the vaginal tissue that affects the tissue strength and causes prolapse. The overall collagen content was found to be lower [47], and the composition of the connective tissue was found to be altered [10,43,48] in patients with prolapse in comparison to those without. It seems as though the fibroblast function, and subsequently, the connective tissue, is already changed due to the increased mechanical load applied to the pelvic tissues. Eventually, these alterations can accumulate on the connective tissue, together with the contribution of the decreasing estrogen levels due to aging, which can lead to the development of POP. Therefore, the answer to the question of “cause and result” for the fibroblasts in POP is leaning more towards “cause”. 

However, some points should be mentioned before making firm conclusions. Making comparisons between studies and drawing concrete conclusions are challenging due to the variation among patients, limited samples sizes, differences in the biopsy sites, or heterogeneity in methods such as protein quantification and histological examinations. In addition, the process of human tissue procurement, which is cross-sectional by necessity, might interfere with the changes observed in the biopsy, such as increased collagen III. 

Even after recognition of these limitations, the role of fibroblasts on connective tissue quality and tissue regeneration, and thereby on the development or treatment of POP, is undeniable. Changes in the collagen metabolism and altered ECM remodeling are a result of altered fibroblasts/(myo)fibroblast behavior due to exposure to mainly mechanical loading, and due to estrogen deprivation or genetic mutations [5,42]. Altered fibroblasts/(myo)fibroblasts’ function may contribute to the impaired wound healing of the pelvic tissues, which subsequently affect the mechanical properties of the tissues that could lead to the incidence or progression of POP. Estrogen seems to improve the mechanical properties of the stretched tissues and could be beneficial for vaginal wound healing. This supports the hypothesis that “POP is a disturbed healing condition”. We suggest that POP interventions should be performed at an early stage, shortly after the mechanical trauma, when the patient’s wound healing and tissue regeneration capacity is still high. Prolapsed tissue already has a lower tolerance for mechanical loading as compared to healthy tissue. The damage in the tissues might increase over time considering the decreasing estrogen levels by age. One explanation of the high recurrence rates of native tissue repairs or the undesired surgical outcomes such as fibrosis after implant surgeries might be untimely operations. 

Future research should focus on regulating (myo)fibroblastic transition and its activity to improve healing. Future studies should be performed under mechanical loading in which fibroblasts from POP and non-POP patients respond differently in terms of collagen metabolism. These studies also provide insight to develop implants with appropriate mechanical properties, in which fibroblasts can contribute tissue regeneration by differentiation into (myo)fibroblasts and by the formation of the appropriate connective tissue. In addition, the activity of (myo)fibroblasts can be regulated by changing the mechanical stress in the environment. Inhibition of the profibrotic cytokines such as TGF-β1 may interfere with the multitude of cells contributing to tissue repair rather than avoiding fibrosis. One potential approach is manipulating (myo)fibroblasts’ mechano-perception via cell-to-matrix and cell-to-cell adhesions to minimize the internal stress, which triggers the apoptosis of (myo)fibroblasts [72]. 

In conclusion, even though we still have a way to go, current advances in understanding the role of fibroblasts in wound healing, fibrosis, and pathophysiology of POP may lead to the prevention of POP and improved surgical outcomes.
